# Functional Properties of Enriched Curd with Collagen and Plant Phytochemicals for Athletes and Physiological Benefits: Evidence Data from Preclinical Trials In Vivo

**DOI:** 10.3390/nu17213373

**Published:** 2025-10-27

**Authors:** Klara Zharykbasova, Aitbek Kakimov, Yerlan Zharykbasov, Zhainagul Kakimova, Raimkhanova Guldana, Kozykenova Zhanna, Beisembayeva Galiya, Zhanat Baigazinov, Tibor Kovács, Amin Shahrokhi

**Affiliations:** 1 Department of Applied Biology, Alikhan Bokeikhan University, Semey 070000, Kazakhstan; 2 Department of Technological Equipment, Shakarim University, Semey 071412, Kazakhstan; 3 Department of Biotechnology, Shakarim University, Semey 071412, Kazakhstan; 4 Department of Physiological Disciplines Named After T.A. Nazarova, NCJSC, Semey Medical University, Semey 070000, Kazakhstan; 5 JSC “Park of Nuclear Technologies”, Kurchatov 071100, Kazakhstan; 6 Research Centre for Biochemical, Environmental and Chemical Engineering, University of Pannonia, 8200 Veszprem, Hungary

**Keywords:** functional foods, collagen-containing concentrate, cottage cheese product, collagen-containing concentrate, sports nutrition, phytochemical extracts, in vivo experiment

## Abstract

**Background/Objectives**: The aim of this study was to establish the multifactorial physiological effect of a functional curd product enriched with collagen-containing concentrate and phytochemical extracts of various natures, under conditions of in vivo experiment. **Methods**: Biomarkers, such as antioxidant activity (glutathione peroxidase, glutathione reductase, MDA), immune response (IgA, IgG, IgM, IL-6, TNF-α), and purine metabolism (uric acid, xanthine oxidase, 5′-nucleotidase) were selected for evaluation and their influence change. The model was white outbred rats (*n* = 45), randomly distributed into three groups: control (basic product), experimental group 1 (supplements of collagen-containing concentrate and extract of the composition of sea buckthorn and rosehips), and experimental group 2 (supplements of collagen-containing concentrate and extract of the composition of yarrow and sage). **Results**: In both experimental groups, a reliable increase in the enzymatic activity of the antioxidant system, a decrease in lipid peroxidation and the level of proinflammatory cytokines, an increase in immunoglobulins, and activation of 5′-nucleotidase were observed. The most pronounced effects were observed with the introduction of a curd product containing collagen-containing concentrate and sea buckthorn and rosehip extract. **Conclusions**: The scientific novelty of the study lies in the first comprehensive in vivo evaluation of the combined enrichment of a dairy product with collagen and plant extracts for a set of biomarkers. The data obtained confirm the physiological activity and functional properties of the developed product, which can be considered as a promising means of specialized and sports nutrition with proven biological action.

## 1. Introduction

From technology to biology, food process engineering provides us with a framework for the development process of functional products that integrates technological innovation with biological efficacy. Therefore, curd-based dairy systems form an effective carrier for bioactive compounds in sports as well as preventive nutrition due specifically and significantly to their structural matrix, nutritional value, and widespread dietary use. Meanwhile, oxidative stress, inflammatory processes, and metabolic dysregulation are still major issues among athletes and humans subjected to high physical loads. The production of curd products with a combination of collagen-containing concentrates and phytochemical extracts provides a process-based approach that enables it to be structurally stable at the same time as it exerts a targeted physiological benefit. Such engineered formulations could bolster antioxidant protection, modulate immune response, and regulate metabolic adaptation to connect technology to biology.

Oxidative stress, inflammatory processes, and metabolic disorders play a key role in the pathogenesis of chronic diseases and the reduction in the body’s adaptive capacity under high physical loads [1,2,3,4,5]. Against the background of these processes, a decrease in antioxidant protection, destabilization of the immune system, and deterioration in the regulation of purine metabolism are observed [6,7,8]. In this regard, functional nutrition is considered an important factor capable of exerting a corrective effect on metabolic and functional disorders [9,10].

Functional food products, including dairy products, are currently considered an effective means of providing the body with biologically active substances. Due to their structural matrix, high nutritional value, and widespread use in the diet of the population, dairy products represent an optimal basis for encapsulation, stabilization, and targeted transportation of biologically active substances, including antioxidants, prebiotics, probiotics, postbiotics, peptides, vitamins, and minerals [11,12,13,14]. Curd products are nutritionally valuable because they are composed of good amino acids and can be added into the diets of individuals who are active. The supplementation of such products with nutrients with proven physiological action widens their functional potential [15,16,17].

Collagen is known as an abundant source of amino acids associated with connective tissue synthesis, regeneration, and general protein metabolism maintenance. Studies show that collagen peptides can have advantages regarding skin, joint, and intestinal barrier health and contribute to immune modulation; modern studies confirm that collagen peptides possess pronounced metabolic and adaptogenic potential. Their consumption promotes an increase in endogenous collagen synthesis, improves body composition, accelerates recovery after physical exertion, and normalizes metabolic processes. Collagen derived from animal by-products serves as a valuable source of bioactive peptides with antioxidant and anti-inflammatory properties that influence amino acid and energy metabolism. Due to its high bioavailability, hydrolyzed collagen can stimulate anabolic processes and maintain the energy balance in muscle tissue [18,19,20,21,22,23]. The combined use of collagen-containing concentrates with plant-based extracts enhances these effects, providing antioxidant protection, tissue regeneration, and metabolic adaptation of the body during physical activity. Furthermore, the synergy between the collagen-containing concentrates and the plant extracts increases the benefits, offering antioxidant protection, tissue regeneration, and metabolic adaptation of the body during exercise. Simultaneously, most fresh investigations also have recognized plant extracts as rich sources of phytochemical compounds—polyphenols, flavonoids, carotenoids, and micronutrients—that have very strong antioxidant and metabolism-controlling effects. Bioactive substances from plants, as stated by the researchers, can boost the body’s antioxidant potential, enhance tissue oxygen uptake, and facilitate energy metabolism, which in turn is directly linked to improved performance and stamina in athletes. The antioxidant compounds are shown to activate the defense enzymes, stabilize the cell membranes, and reduce muscle tissue damage caused during vigorous exercise. The use of phytochemical substances for enriching dairy products is a new opening for functional foods with pronounced physiological effects [24,25,26]. Thus, the use of plant-derived extracts in combination with a collagen-containing concentrate strengthens antioxidant defense, promotes the restoration of cellular metabolism, and enhances the adaptive capabilities of the athlete’s body.

While the combination integration with collagen and phytochemicals has powerful physiological effects, such as stimulation of tissue regeneration, diminishment of oxidative stress, and anti-inflammatory action, these pathways are seldom studied as a complementary approach in vivo. For example, Xu et al. developed a ternary complex of un-denatured type II collagen, a hydrophobic phytochemical, and chondroitin sulfate, that showed excellent stability and the selective delivery of an active substance into the intestine and is expected to play the role of a candidate for osteoarthritis treatment. At the same time, Hanga-Farcaș et al. (2023) stresses that despite the promising role of phytochemicals for bone tissue regeneration, the implementation of most innovative means for its delivery collateral matrices and nanostructures continues to be the object of study and is still waiting for in vivo validation [27,28]. At the same time, there is a lack of data on the systematic assessment of functional effects of dairy products enriched with both collagen and plant antioxidants using biomarkers of antioxidant status, immune activity, and validated purine metabolism. That limits their use in sport and therapeutic and prophylactic nutrition.

This is supported by the review of Guerriero et al. (2025) [29], in which 61 studies among students were analyzed and a coherent positive relation was established between the level of physical activity and the reduction in manifestations of stress. In our work, the changes in enzymatic activity and immunological markers might be considered not only from the sportive point of view but also from the wide physiological and psychophysiological standpoint.

It is interesting to note that functional effects accompanying enzymatic and immune regulation could be positive not only in the cases of actively training but also in sedentary or chronically stressed groups.

The issues pointed out in the review—for example, varied experimental protocols and the shortage of generally accepted methods—correspond to our findings about high interindividual variability and the low sample size. It shows the need for further research with strictly defined parameters (type, duration, and intensity of physical activity) and detailed assessment of the combined effects of nutrition, physical activity, and stress regulation [29].

Paying attention to the proven metabolic and antioxidant effects of collagen-containing peptides and phytochemical compounds, it can be assumed that enrichment of the curd product with a combination of collagen-containing concentrate and antioxidant plant extracts contributes to the modulation of key metabolic processes, including a decrease in the level of oxidative stress, an increase in the humoral immune response, and the regulation of the enzymatic activity of purine metabolism. Such a complex effect can indirectly contribute to the formation of a pronounced physiological response of the body to physical activity. Given the insufficient study of such biocompatible food compositions under in vivo, this study has scientific novelty and emphasizes the potential of the developed product as a promising means of functional and specialized sports nutrition.

The aim of this study is to conduct a comprehensive in vivo assessment of the functional properties of a curd product enriched with collagen and plant extracts, in terms of antioxidant protection, humoral immunity, and purine metabolism.

## 2. Materials and Methods

### 2.1. Object and Methods of Research

The object of the study was functional curd products enriched with collagen-containing concentrate and plant extracts, intended for specialized sports nutrition. The technology for obtaining collagen-containing concentrate, antioxidant plant extract, as well as the technology for obtaining curd products, are described in detail in our previous work [30].

To obtain the dry collagen-containing concentrate, secondary poultry processing products—skin, bone tissue, and feet—purchased from retail outlets were used as the raw material. A gel concentrate was produced from chicken skin. The chicken skin was cut into fragments 2–3 cm long, followed by heat treatment at 65 °C for 3 h and subsequent cooling to 36 °C. It was treated with papain enzyme at a ratio of 1:10 and enzymatic hydrolysis was performed at 36 °C for 24 h. The resulting mixture was cooled down to 20–25 °C, kept for 60 min, centrifuged at 1000 rpm, and the obtained gel was finally taken out and cooled to 5–6 °C.

In parallel, a paste-like collagen-containing concentrate was prepared from a mixture of bone tissue and chicken feet (in a 40:60 ratio). The raw material was heat-treated at 65 °C for 3–4 h, ground to 2–3 mm, papain was added (1:10), and enzymatic hydrolysis was carried out at 36 °C for 24 h. The resulting concentrate was then cooled to 5–6 °C.

The obtained gel and paste-like collagen-containing concentrate were mixed in a 1:1 ratio and thoroughly stirred for 15–20 min. The resulting mixture was subjected to freeze-drying at −44 °C for 18 h until a final moisture content of 7–8% was achieved. Standardization of the obtained dry collagen-containing concentrate was performed based on the collagen content in dry matter (55.4%) and solubility degree (92.6%).

To obtain the plant extracts, fruit and berry raw materials (sea buckthorn Hippophae rhamnoides and cinnamon rosehip Rosa majalis) and medicinal plant materials (common yarrow Achillea millefolium and steppe sage Salvia stepposa) were used, collected during field expeditions in the Abai Region of the Republic of Kazakhstan. Extraction of plant material was carried out at 25 °C for 3 h with stirring at 175 rpm. For fruit and berry raw materials, 75% ethanol was used at a ratio of raw material:solvent = 1:5. For medicinal plant material, 96% ethanol was used at a ratio of 1:10. The difference in ethanol concentration was determined by the polarity of the extracted compounds. The same 75% ethanol provided the highest values of flavonoid content for the fruit and berry raw materials—up to 95.1 mg/100 g—and a high quercetin level of 9.4 mg/100 g, whereas the content of vitamin E was somewhat lower (2.3 mg/100 g), compared to 96% ethanol. Medicinal plant materials were best extracted by 96% ethanol to give the following values: flavonoids at 112.7 mg/100 g, vitamin E at 3.1 mg/100 g, and quercetin at 11.6 mg/100 g. Standardization of the extracts was performed considering total polyphenols and flavonoids content expressed in gallic acid and quercetin equivalents, respectively. Mean values of 42.8 ± 1.7 mg GAE/g and 17.2 ± 0.8 mg QE/g provide evidence for the high antioxidant potential of the obtained extracts.

Experimental samples of the curd product were produced using the following technology: Pasteurized skim cow’s milk (acidity no higher than 18 °T) was used as the main raw material. Fermentation was carried out at a temperature of 30–32 °C for 6–8 h until a titratable acidity of 71–73 °T (pH = 4.5 ± 0.05) was reached using 5% starter culture based on mesophilic and thermophilic lactic acid streptococci, sodium chloride (400 g/t), and 1% rennet enzyme (Chr. Hansen, Denmark) based on chymosin (≈95% chymosin and ≈5% bovine pepsin). After curd coagulation and pressing, the curd was ground to a uniform consistency and enriched with a dry collagen-containing concentrate (8%) and a plant-derived extract (4%). After cooling, the product was stored at 4 ± 2 °C.

According to the developed technology, three types of low-fat curd product samples were prepared under laboratory conditions for the study:

Control sample—without the addition of functional ingredients;

Experimental sample 1—with the addition of 8% collagen-containing concentrate and 4% extract composed of sea buckthorn (Hippophae rhamnoides) and cinnamon rosehip (Rosa majalis);

Experimental sample 2—with the addition of 8% collagen-containing concentrate and 4% extract composed of common yarrow (Achillea millefolium) and steppe sage (Salvia stepposa).

### 2.2. Experiment In Vivo

The biological effects of these products were assessed under in vitro conditions in vivo on laboratory animals.

For the in vivo experiment, white mongrel rats (n = 45), weighing 180–220 g and aged three months, were selected (the rats were provided by the Research Center of the Non-Commercial Joint Stock Company Semey Medical University, Semey, Kazakhstan). The animals were randomized into three groups:

- Control group (n = 15), which received a basic curd product without fillers;
- Experimental group 1 (n = 15), which was administered a functional product enriched with collagen and fruit and berry plant extract (sea buckthorn and cinnamon rose hips);
- Experimental group 2 (n = 15), which was administered a functional product enriched with collagen and medicinal plant extract (yarrow and sage).

The product was administered orally twice daily at a dose of 2 g for 7, 14, and 21 days. The first group of animals received the products per os for 7 days according to the experimental scheme, after which they were euthanized. The obtained materials (serum and plasma) were evaluated depending on the research method. The study was also continued in the experimental groups for 14 and 21 days. No treatment was administered.

All studies related to the maintenance and experiments on laboratory animals were carried out in strict accordance with the provisions of the European Convention for the Protection of Vertebrate Animals used for Scientific Purposes (ETS No. 123), as well as in accordance with the legislation of the Republic of Kazakhstan. Ethical review of the study was carried out by the Local Ethics Commission of Semey Medical University. At meeting No. 2 dated 7 November 2022 (Protocol No. 2), the project “Scientific and practical basis for the use of collagen-containing concentrate in the production of specialized curd products for athletes’ nutrition” was considered.

### 2.3. Research Methods

The study included the use of immunological, biochemical, and enzymatic methods of analysis aimed at a comprehensive assessment of antioxidant activity, levels of inflammatory and immunological markers, as well as the state of purine metabolism.

#### 2.3.1. Immunological Indicators

Solid-phase “sandwich” ELISA method with monoclonal antibody-based test kits for light Ig chains (λ and κ), “Immunoglobulin A-ELISA-BEST,” “Immuno-globulin M-ELISA-BEST,” and “Immunoglobulin G-ELISA-BEST” (Vector-Best Joint Stock Company, Novosibirsk, Russia) were used to determine immunoglobulin levels (IgA, IgM, IgG).

Blood samples were collected from each animal into sterile dry tubes in a volume of no less than 1.5–2 mL and placed in a thermostat at 37 °C for 1 h. The resulting blood clot was gently detached with a sterile metal needle or glass rod and then placed in a refrigerator (+4 °C) for 1–2 h to allow the serum to separate.

Modern enzyme-linked immunosorbent analyzers (ELISA systems) are automated units that perform reagent mixing, sample movement, mixer and dispenser cleaning, incubation of test samples, and system sterilization, thereby enabling a complete analysis cycle. The sequence of all analytical stages is controlled by dedicated software. Optical density was measured at 450 nm with a reference wavelength of 620–655 nm. The results were calculated using a calibration curve [31,32,33].

#### 2.3.2. Determination of Proinflammatory Cytokines (TNF-α, IL-6)

The procedure was similar, using ELISA kits (Cloud-Clone Corp., Wuhan, China) specifically designed for TNF-α and IL-6 determination. In response to stress, including physical exertion, the body releases cytokines. These substances trigger a cascade of reactions leading to increased production of glucocorticosteroids (e.g., cortisol) and a decrease in immune cell activity. Cortisol suppresses the production of proinflammatory cytokines (IL-6, TNF-α) during physical exercise. However, the sensitivity of these cytokines to cortisol varies: TNF-α is inhibited even by physiological doses of cortisol, whereas IL-6 remains resistant to its effects.

This difference in sensitivity explains why IL-6 levels often rise sharply and remain elevated after physical activity. The increase in IL-6, in turn, stimulates the production of “acute-phase” proteins, which can induce inflammation, including that associated with muscle damage during training.

When comparing different types of physical exercise, it has been found that an in-crease in IL-6 concentration and enzymes indicative of muscle damage (creatine kinase, aspartate aminotransferase, alanine aminotransferase) occurs only during eccentric exercises (when a muscle is stretched under load) [34].

The analysis was performed in accordance with the manufacturer’s instructions, with measurements taken at 450 nm [35,36].

#### 2.3.3. Biochemical Indicators of Antioxidant Activity

The level of malondialdehyde (MDA), used as a marker of lipid peroxidation, was determined spectrophotometrically after reaction with 2-thiobarbituric acid (λ = 520 nm) [37]. MDA is a degradation product of fatty acids formed as a result of oxidation. The amount of MDA reflects the intensity of the processes of lipid oxidation. Malondialdehyde at elevated temperatures reacts with 2-thiobarbituric acid to form a colored complex having maximum absorption at 520 nm. The molar extinction coefficient (E) of this complex is 1.56 × 10^5^ M^−1^·cm^−1^, and the results are expressed in nmol per mg of total lipids [38]. To determine the content of diene conjugates (DC), the light absorption of the extract of lipids in the UV region was measured at 233 nm [39]. Conjugated dienes are compounds formed under the conditions of free radical oxidation of polyunsaturated fatty acids from double bonds rearrangement. When a hydrogen atom is abstracted from the PUFA molecule, the carbon atom with an unpaired outer-shell electron acquires sp^2^ hybridization. The emerging non-hybridized p-orbital comes into conjugation with the p-orbitals of neighboring sp^2^-hybridized carbon atoms. Thus, a system of conjugated double bonds arises within the PUFA molecule, that is, a diene conjugate [40].

The activity of glutathione peroxidase and glutathione reductase was evaluated based on the enzymatic transformation of glutathione, with measurements taken at wavelengths of 260 nm and 340 nm, respectively [41]. Glutathione peroxidase is one of the main enzymes of the antioxidant defense system, and it represents the class of oxidoreductases being widely distributed in cells, including endothelial cells. The enzyme contains four identical subunits, and the active site thereof is enriched with selenium. Such an enzyme uses reduced glutathione (GSH) as a substrate and oxidizes it into glutathione disulfide (GSSG) during the catalyzed reaction. Glutathione disulfide is reduced back to GSH by glutathione reductase. Thus, the glutathione enzyme system is part of cellular homeostasis maintenance. GPx ensures both the formation and regeneration of reduced glutathione for the continuous function of the glutathione peroxidase system [42].

Glutathione reductase catalyzes the reduction of oxidized glutathione, using NADPH_2_—produced in the pentose phosphate pathway during glucose oxidation—as hydrogen (electron) donor.

Catalase activity was measured according to the decrease in optical density of hydrogen peroxide solution during its complex formation with sodium molybdate (λ = 410 nm) [43]. The main enzymes that would prevail in this system are superoxide dismutase (SOD), catalase, and glutathione-S-transferase (GST). SOD plays a very significant role in neutralizing the superoxide anion radical and preventing cellular damage under conditions of oxidative stress. Catalase scavenges hydrogen peroxide formed as a result of SOD activity. GST, in turn, reduces negative effects of oxidative stress and toxins by restoring damaged lipids in cell membranes, using glutathione. GST also takes part in elimination of toxic products of lipid and protein oxidation from the organism by its conjugation with glutathione.

These antioxidant mechanisms, in turn, are interconnected in a manner that sustains the redox homeostasis of an organism. Enzymatic and non-enzymatic antioxidants, acting in concert, are resistive to stress factors promoting the generation of ROS and free radical oxy-oxidation.

#### 2.3.4. Purine Metabolism Indicators

The activities of 5′-nucleotidase, adenosine deaminase, and AMP deaminase were determined colorimetrically using a modified Berthelot reaction for the quantitative measurement of ammonia (λ = 540 and 840 nm) [44].

#### 2.3.5. Statistical Analysis

Statistical processing of the results was performed using nonparametric methods. Since the distribution of most variables did not correspond to normal, the Kruskal–Wallis criterion was used to assess the differences between three independent groups. The data are presented as median with interquartile range (Q1–Q3). The level of statistical significance is set at *p* < 0.05. All calculations were performed in SPSS 20.0 software.

## 3. Results and Discussion

At the first stage, studies were conducted under in vivo conditions with antioxidant indices of curd product samples. The results are presented in Table 1. The content of diene conjugates, the level of malonic dialdehyde (MDA), as well as the activity of key antioxidant enzymes (catalase, glutathione peroxidase, and glutathione reductase), reflect the product’s ability to reduce oxidative stress levels and thereby prevent damage to cellular structures.

Detailed pairwise analysis (Table 2) confirmed that the enriched curd product significantly modulated antioxidant enzymes and lipid peroxidation markers under in vivo conditions.

As shown in Table 1 and Table 2, animals in experimental group 2 showed a significant increase in glutathione reductase activity compared to the control group (*p* = 0.002) and between the two experimental groups (*p* = 0.046). The intergroup differences were of moderate to strong magnitude, which may indicate an increased ability of the body to regenerate reduced glutathione and, as a result, to neutralize free radicals. An increase in the activity of this enzyme can be regarded as a manifestation of increased redox protection caused by the action of biologically active substances of the functional ingredients of the experimental samples of the curd product. According to the results of Yang et al. (2006) [45], the activity of glutathione reductase is one of the key factors determining the resistance of cells to oxidative stress. It is this enzyme that ensures the restoration of glutathione, which is involved in the detoxification of peroxide compounds. Thus, the increase in the activity of glutathione reductase that we recorded may indicate an increase in the antioxidant protection of the body against the background of consumption of a product enriched with a collagen-containing concentrate and plant extracts [45].

At the same time, a decrease in glutathione peroxidase activity was observed in both experimental groups compared to the control group (*p* ≤ 0.05), with strong inter-group differences (r ≈ 0.5). This is probably due to the redistribution of the functional load between the components of the glutathione-dependent link of antioxidant protection, where the increased activity of glutathione reductase compensates for the need for the inactivation of hydroperoxides. Such a pattern may indicate a transition from the phase of acute oxidative stress to a state of adaptation, when the need for activation of the “first line” enzymes decreases. Such interdependence of glutathione peroxidase and glutathione reductase activity is also pointed out by Yang et al., 2006 where, using examples, it was shown that a decrease in the activity of one of these enzymes can be compensated by increasing the other, reflecting the ability of adaptive flexibility and balance of the work of the antioxidant system of cells in response to external stress factors [45].

Catalase activity indices of the control and experimental groups did not have statistically significant differences either between the experimental and control groups or between the experimental groups among themselves (*p* ≥ 0.05), which testifies to stability of this component of the antioxidant system in studied conditions. It is likely that catalase performs a baseline protective function without being actively involved in compensatory mechanisms when the tested products are used.

The level of malondialdehyde (MDA), a reliable marker of lipid peroxidation, de-creased significantly in the first experimental group compared to the control (*p* = 0.021)—by 23.8% (from 0.340 ± 0.015 to 0.259 ± 0.012 nmol/mg of protein). In experimental group 2, a comparable but less noticeable pattern was noted. This suggests that the degree of damage caused by free radicals to the lipid components of cell membranes has decreased. This could be interpreted as the reduction in the intensity of free radical damage to lipid components of cell membranes. Therefore, the antioxidant activity of the complex containing collagen and phytochemical compounds, which likely inhibit lipid peroxidation processes, as a result increases cellular resistance to oxidative stress. Similar protective effects of collagen-containing and plant-derived components have also been reported in several other studies [46,47,48], while the role of MDA as a key marker of oxidative damage is confirmed in the fundamental reviews by Rahal et al. (2014) [49].

The dynamics of the level of diene conjugates (DC) is of considerable interest. In both experimental groups, their content significantly exceeded the values of the control group (*p* = 0.004, *p* = 0.013), which may indicate the activation of protective and adaptive processes in the body. An increased level of DC with a simultaneous decrease in the concentration of malonic dialdehyde (MDA) suggests inhibition of chain reactions of lipid peroxidation at early stages. Such changes may indicate the formation of metabolic adaptation—a condition characterized by the activation of endogenous regulatory systems that contribute to increased resistance of the body to oxidative stress.

Thus, the identified changes in biochemical markers of antioxidant status indicate a positive effect of the fortified curd product on maintaining the redox balance in the body. The data obtained suggest that the inclusion of collagen-containing and phytochemical components in the diet contributes to the formation of adaptation mechanisms to oxidative stress, which occurs, among other things, during physical exertion. These effects reflect the ability of the product to reduce the level of oxidative stress and thereby prevent damage to cellular structures.

At the next stage, in vivo studies of immunological parameters of experimental samples of curd product were carried out. Determination of levels of immunoglobulins (IgA, IgM, IgG) and proinflammatory cytokines (TNF-α, IL-6) showed the presence of a potential modulating effect on the humoral link of immunity and the inflammatory response, as shown in Table 3.

The results of in vivo studies demonstrated a regulatory influence of curd products containing collagen and/or plant extracts on laboratory animals’ humoral immunity indices and proinflammatory cytokine concentrations. The most prominent changes were seen in the circulating levels of immunoglobulins of classes A, M, and G. Although these IgM and IgG levels did not reach statistically significant differences, significant reductions in IgA levels were noted in the experimental groups, as well a trend towards greater circulating IgM and IgG levels. Overall TNF-α and IL-6 proinflammatory cytokine concentrations levels did not reach statistically significant differences between groups. More detailed intergroup comparisons can be found in Table 4.

In the initial experimental group, where the product was enhanced with collagen and sea buckthorn and rosehip extracts, a statistically significant reduction in IgA concentration was observed when comparing to the control group (*p* = 0.001, r = 0.43). Given that IgA is an essential component of mucosal immunity and acts as a defense mechanism for the mucous membranes of the respiratory tract and gastrointestinal tract, this change in concentration could indicate that the product may modulate an aspect of the local immune response. This possible modulation is likely due to the bioactive phytochemical components contained in the fruit and berry extracts. In the second experimental group that contained yarrow and sage extracts, the level of IgA did not differ from control (*p* = 0.84), indicating a more buffered, immunomodulating effect of the plant extracts, not producing an overall immunomodulating effect or at least a very limited effect on secretory IgA levels. The IgM level (an immunoglobulin that is responsible for the pre-dominantly humoral immune response) tended to be higher in the first experimental group and was statistically significantly different (*p* = 0.03, r = 0.47). Such a result may indicate an initial activation of the immune system under the influence of the functional components of the product. A similar trend was observed for IgG, an antibody associated with the formation of long-term immune response. In both experimental groups, the concentration of IgG tended to increase compared to the control group; at the same time, in experimental group 1, intergroup comparison showed statistically significant differences (*p* = 0.024, r = 0.48), which reflects the involvement of secondary mechanisms of humoral immunity and the formation of a more prolonged adaptive response. In general, the other comparison groups did not show statis-tically significant differences. This may indicate an enhancement of the body’s defense mechanisms and activation of the adaptive branch of the immune system.

Different levels of proinflammatory cytokines (TNF-α and IL-6) did not significantly differ between groups. TNF-α level remained stable (*p* = 0.978) during testing, suggesting there was no systemic inflammation during the consumption of the product. Concurrently, IL-6 level had more of a tendency to decrease from baseline, which was more pronounced for the second experimental group (*p* = 0.079). This may indicate the anti-inflammatory effect of plant components and the activation of metabolic adaptation mechanisms. Moderate IL-6 reduction within the physiological range is often associated with the stabilization of the inflammatory background and participation in tissue regeneration and metabolic processes. Shahbazi et al. (2021) have previously reported comparable dynamics in that fermented plant products can promote the decrease of proinflammatory cytokines (such as IL-6 and TNF-α) because of their bioactive compounds (like polyphenols and organic acids) and support the restoration of immune homeostasis [50]. Thus, our results are consistent with the literature’s evidence regarding the mild anti-inflammatory effect of plant ingredients in functional food products.

The data obtained confirm that the curd product developed exerts a regulatory, directed effect on the immune system of the organism, activating certain components of humoral immunity without evidence of inflammatory hyperactivation. These effects are particularly important in conditions with high physical activity (showing effects in athletes, for example). Similar mechanisms were described by Tian et al. (2021) [51], and they stated the role of natural immunomodulatory compounds (polysaccharides, polyphenols) on mediating immune response, especially under conditions of allergy. Our findings, although based on a different experimental model, corroborate the immunomodulatory influence of biologically active substances of plant origin [51]. Finally, Singh et al. (2023) [52] emphasized the impact of the regular consumption of foodstuffs containing natural antioxidants and nutrients on immune homeostasis. This is in full agreement with our evidence of a mild stimulating effect of the developed product without stimulant-induced activation of the inflammatory response [52].

Consequently, the current study contributes to and improves the theoretical basis of the role of functional nutrition in the regulation of immune response, providing evidence in an in vivo model that a curd product containing collagen and plant extracts can serve as an effective component of a potential diet with some immunomodulatory potential. At the end of the study, an in vivo evaluation of the activity of enzymes involved in purine metabolism, 5′-nucleotidase, adenosine deaminase, and AMP deaminase, was carried out. These enzymes are important regulators of adenine nucleotide catabolism, which are compounds involved in the production and utilization of energy in the cell. Their activity represents a state of energy metabolism and ability to adapt to physical stress [53,54,55]. Changes in the activity of these enzymes make it possible to assess how the body processes and uses energy, as well as the influence of the functional product on metabolic processes (Table 5).

**Figure 1:**
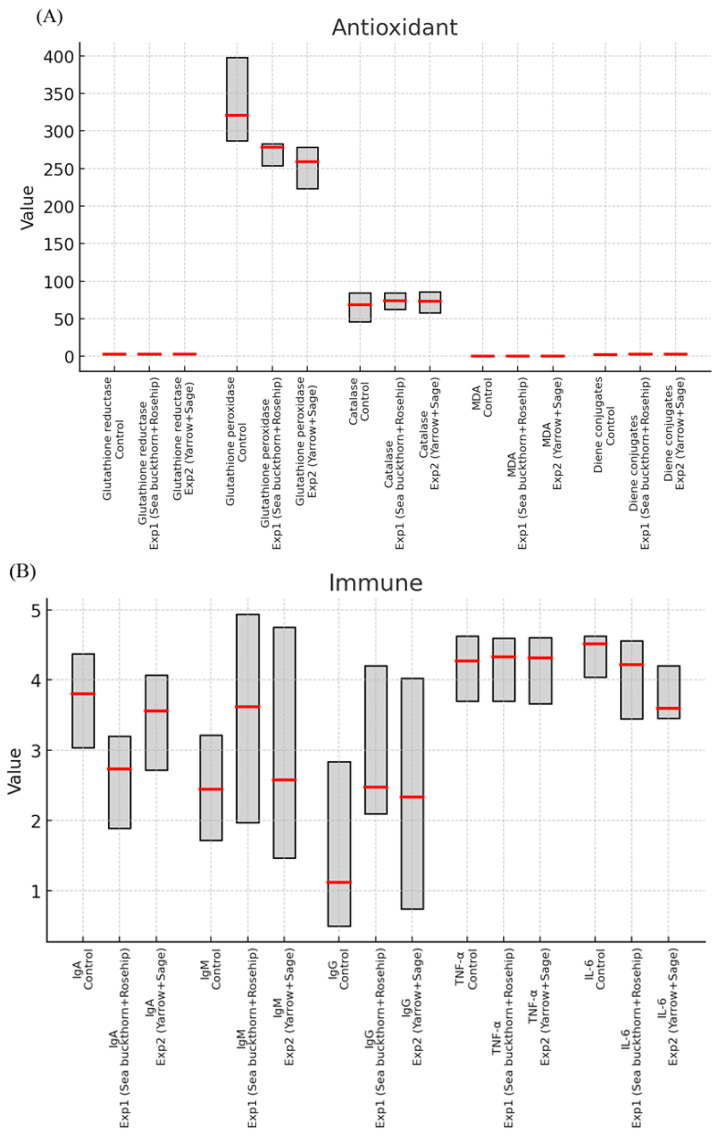

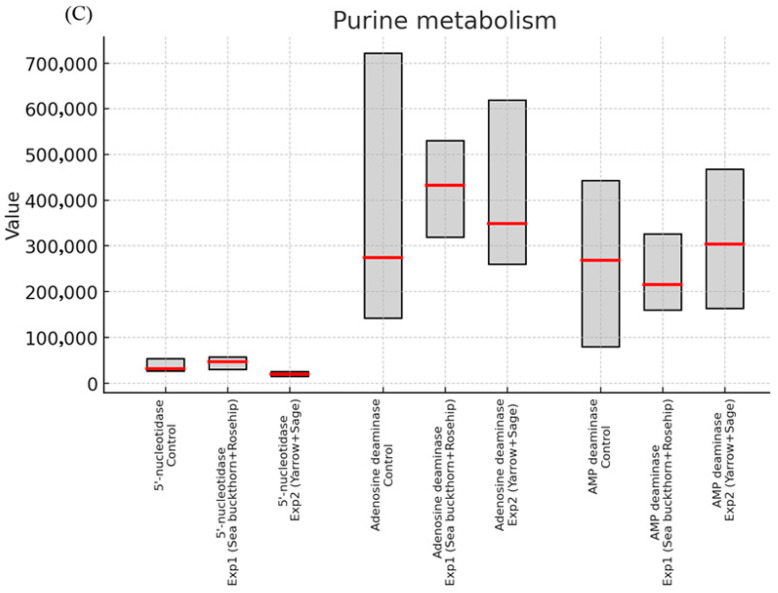
Boxplot-style visualization (Red line marks the median, and the gray box shows the interquartile range (IQR)). (**A**) Antioxidant markers showed that both groups had higher levels of glutathione reductive activity and lower levels of glutathione peroxidase compared to controls, while catalase remained constant; meanwhile, MDA showed a selective reduction in group 1 and diene conjugates increased in both groups. (**B**) Immune markers of IgA decreased significantly in group 1, while IgM and IgG both increased; TNF-α remained stable and IL-6 appeared to decrease in group 2. (**C**) Purine metabolism enzymes displayed that 5′-nucleotidase increased in group 1 and decreased in group 2, while adenosine deaminase and AMP deaminase did differentiate.

In addition to showing results in tables, Figure 1 provides biomarker (median and interquartile ranges) distributions among participants in a boxplot style for each group. The box and whisker plot delineates the metrics and the variation about them and proportions of the data to each other, which augments the needed statistical analyses and tells us how the experimental formulations differ from the control values.

The study showed that a curd product rich in collagen and plant extracts affects the activity of enzymes associated with the metabolism of purines—substances involved in energy production in the body. The most noticeable alterations were noticed in the functioning of the enzyme 5′-nucleotidase. For the first experimental group of animals, where the product contains sea buckthorn and rose-hip extract, the activity of this enzyme was found to be much higher than that of the control group (*p* = 0.001). This may indicate a greater synthesis of adenosine, which is an important molecule for cellular protection and energy homeostasis—particularly in response to exercise.

Similarly, in the second experimental group, where yarrow and sage extract were applied, there was less 5′-nucleotidase activity compared to the control. This is probably due to other mechanisms of action of the biologically active substances contained in these plants. According to literary data, individual flavonoids and terpenoids contained in yarrow and sage inhibit COX enzymes and reduce the production of proinflammatory mediators such as prostaglandin E_2_, exerting anti-inflammatory and immunomodulatory effects [56,57,58]. These effects can indirectly improve energy metabolism by reducing inflammation and activating antioxidant protection.

The activity of an additional enzyme (adenosine deaminase) was not significantly different across study groups (*p* = 0.553), whereas in the first experimental group a tendency to increase was observed. This might mean that against the background of activation of adenosine metabolism, the body increases its processing into inosine, another substance involved in energy metabolism and having protective functions.

Another enzyme, AMP deaminase, showed no significant differences in activity from group to group (*p* = 0.330). Yet, a marginal increase in its level was observed in the second experimental group, possibly due to a requirement to process excess AMP, which builds up during intense muscle work.

**Figure 2:**
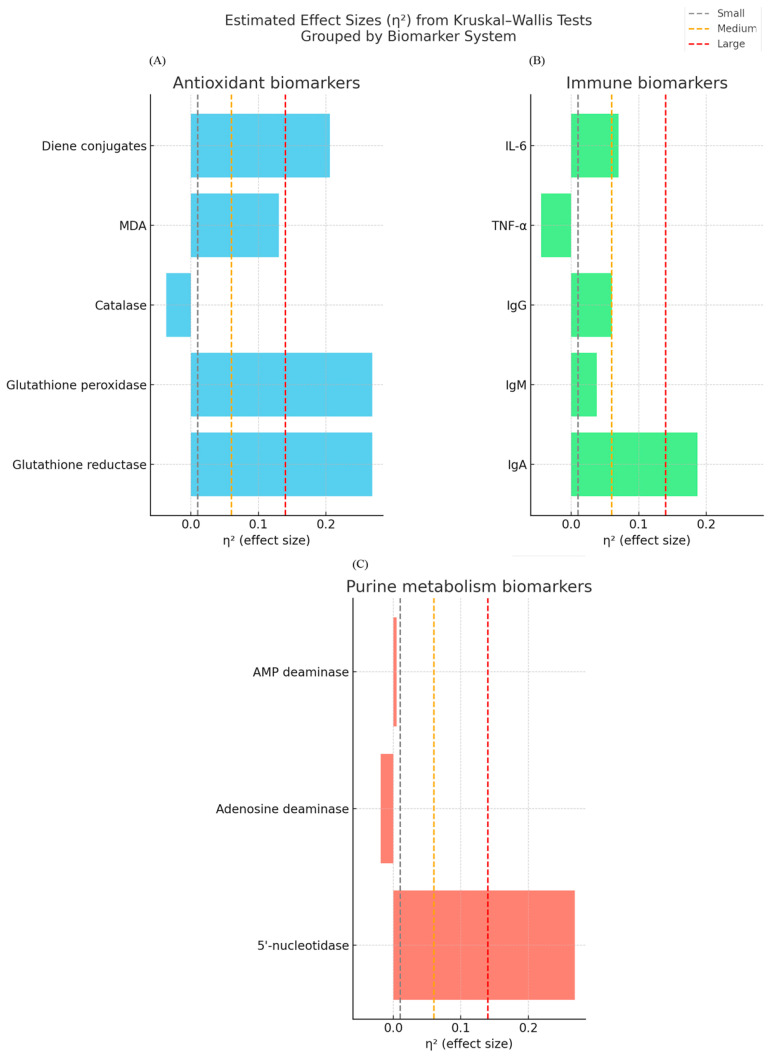
Effect sizes (η^2^) of biomarkers involved in antioxidant, immune, and purine metabolism from Kruskal–Wallis tests. Reference lines are plotted at small (0.01) medium (0.06) and large (0.14) effect thresholds. (**A**). Antioxidant markers. All experimental groups had higher glutathione reductase activity and lower glutathione peroxidase compared to controls, while catalase did not differ. MDA was decreased in group 1 only while diene conjugates were increased in both groups. (**B**). Immune markers. IgA was significantly decreased by group 1, IgM and IgG generally increased, TNF-α remained stable, and IL-6 was trending towards decrease in group 2. (**C**). Purine metabolism enzymes. 5′-nucleotidase activity was increased in group 1 and decreased in group 2, while there was no apparent difference in adenosine deaminase or AMP deaminase.

Moreover, to enhance the tabulated data, the effect sizes (η^2^) of all biomarker comparisons are estimated and shown in Figure 2. This visualization also reveals the strongest physiological effects of enriched curd products on the glutathione-dependent antioxidant system and 5′-nucleotidase activity, whereas the immune modulation effects were selective. Effect sizes were calculated from nonparametric group comparisons (n = 15 each group), where η^2^ values were shown in each panel with reference lines for small (0.01), medium (0.06), and large (0.14) effects. Within the antioxidant system, significant effects of glutathione reductase (η^2^ ≈ 0.27) and glutathione peroxidase were found, as well as the effect of diene conjugates (η^2^ ≈ 0.21), with MDA reaching medium effect (η^2^ ≈ 0.13) and catalase activity negligible. IgA had a large (η^2^ ≈ 0.16) effect among its immune markers, whereas IgM, IgG, TNF-α, and IL-6 were found to be within negligible to small range. 5′-nucleotidase had a major effect in purine metabolism (η^2^ ≈ 0.27) as compared with adenosine deaminase and AMP deaminase, which were almost non-significant. The inclusion of effect sizes beside *p*-values therefore gives a richer picture of the biological relevance of the observed changes.

**Figure 3:**
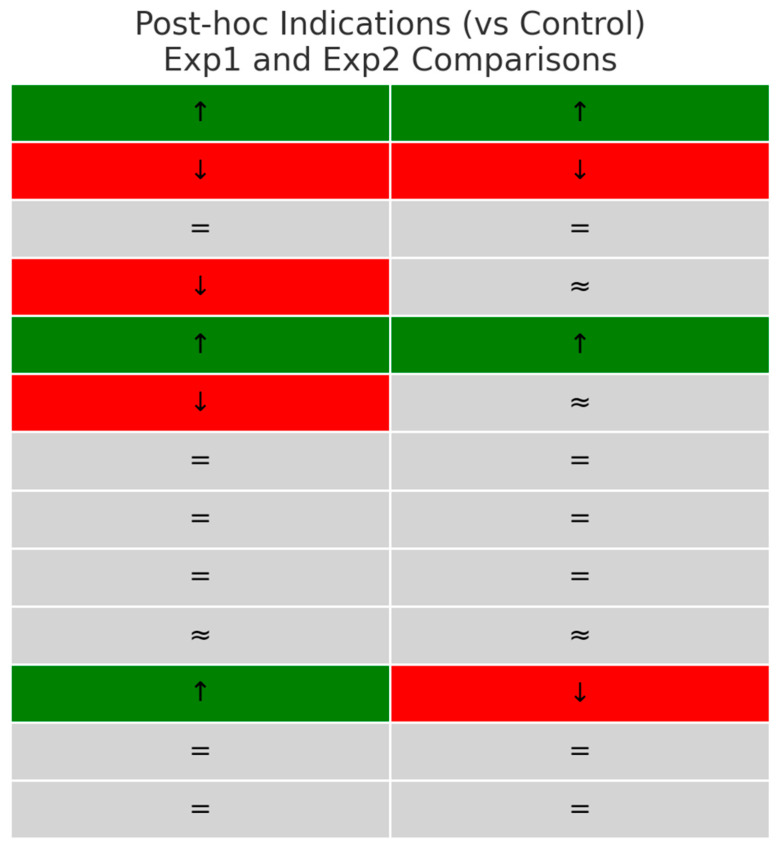
Post hoc indications of differences between experimental groups and control for key biomarkers. (Color coding illustrates the direction of change relative to the control group: green (↑) = higher, red (↓) = lower, grey (= or ≈) = no reliable difference.).

Post hoc inspection of group medians (Figure 3) suggested that the significant Kruskal–Wallis results were mainly driven by the following:

- A higher glutathione reductase and lower glutathione peroxidase activity than that in the control group in both experimental groups;
- Experimental group 1 had reduced MDA levels in comparison to control;
- Elevated diene conjugates in both experimental groups compared with control;
- A selective reduction in IgA in experimental group 1 compared to control;
- Variations in 5′-nucleotidase activity, experimental group 1 greater than the control group and experimental group 2 less than controls.

There were no clear pairwise differences observed for catalase, IgM, IgG, TNF-α, IL-6, adenosine deaminase, or AMP deaminase.

In line with the patterns shown in effect sizes (Figure 2 and Figure 3), enzymes of purine metabolism showed selective modulation: 5′-nucleotidase activity increased substantially in experimental group 1 and decreased in experimental group 2, but adenosine deaminase and AMP deaminase remained stable. This pattern indicates extract-dependent regulation of energy-related pathways without broad shifts in purine turnover.

Overall, our data suggests that the functional curd product has an influence on the metabolism of purines and energy equilibrium in the body. Particularly striking was an effect observed in animals that received the product along with extracts of fruit and berry plants which indicates its adaptogenic and metabolically active effects. This preclinical in vivo study reveals that the addition and enrichment of curd products is a multifactor physiological process. Both experimentations increase enzymatic activity of the antioxidant system, decrease lipid peroxidation, and modulate immune and metabolic markers prepared, in comparison to control. Of note, cross-sectional analysis of effect sizes (η^2^) with respect to all measured parameters (Figure 2) demonstrated that, over all measures, the maximum effects were observed by the glutathione-dependent antioxidant system, and 5′-nucleotidase activity and the immune responses showed a more selective and mild modulation (most markedly, IgA reduction). The results showed that the curd products demonstrated much physiological activity and may be used as functional foods for athletes and specialized nutrition.

## 4. Conclusions

Results of the complex in vivo studies have shown that a curd product enriched with collagen-containing concentrate and antioxidant plant extracts has a multi-profile positive effect on key biomarkers of antioxidant protection, immune response, and purine metabolism. It has been established that consumption of the developed product promotes the activation of glutathione system enzymes, a decrease in the level of malonic dialdehyde (MDA)—a marker of lipid peroxidation, and also modulates the level of immunoglobulins and proinflammatory cytokines. The most pronounced effects were noted in the group receiving the product with sea buckthorn and rosehip extract, which indicates the high biological activity of this phyto-composition. In addition, a reliable increase in the activity of 5′-nucleotidase was established, which reflects the potential of the product in maintaining energy homeostasis during physical exertion. The scientific novelty of this study lies in the first comprehensive in vivo evaluation of a curd product, simultaneously enriched with collagen-containing concentrate and phytochemical extracts of various natures, according to a set of biomarkers of antioxidant status, immune response, and purine metabolism. For the first time, it was experimentally shown that biologically active components in the dairy product have a targeted physiological effect with regular consumption. Thus, the developed enriched curd product can be considered as a promising means of specialized nutrition for people experiencing increased physical activity, including athletes. The data obtained confirm its functional properties and contribute to the development of scientific foundations for the creation of sports and preventive nutrition products with proven physiological activity. The results of the study justify the feasibility of conducting subsequent clinical trials with the participation of target groups of consumers to clarify the mechanisms of action and evaluate the long-term effectiveness of the product.

## Figures and Tables

**Table 1:** Antioxidant activity in rats fed with fortified curd product: comparative analysis.

Indicator	Control Group (Me; Q1–Q3)	Experimental Group 1 (Me; Q1–Q3)	Experimental Group 2 (Me; Q1–Q3)	*p*-Value
Glutathione reductase, U/mg protein	2.58 (2.25–3.01)	3.00 (2.00–3.00)	3.00 (3.00–3.00)	0.001
Glutathione peroxidase, U/mg	321 (287–398)	278 (254–283)	259 (223–278)	0.001
Catalase, U/mg	68.35 (45.68–84.66)	73.65 (62.54–84.19)	73.25 (58.00–86.09)	0.826
Raspberry dialdehyde (MDA), nmol/mg	0.340 (0.277–0.433)	0.259 (0.254–0.321)	0.333 (0.301–0.424)	0.021
Diene conjugates (DC), U/mg	1.90 (1.20–2.00)	2.55 (2.35–2.82)	2.53 (2.07–2.71)	0.004

**Table 2:** Antioxidant activity in rats after inclusion of the enriched curd product in the diet: pairwise group analysis.

Parameter	Control Group—Experimental Group 1	Control Group—Experimental Group 2	Experimental Group 1—Experimental Group 2	*p*-Value
Glutathione reductase, U/mg protein	*p* = 0.075 r = 0.32	*p* = 0.002 * r = 0.56	*p* = 0.046 * r = 0.36	0.001
Glutathione peroxidase, U/mg	*p* = 0.0059 * r = 0.568	*p* = 0.0110 * r = 0.534	*p* = 0.389 r = 0.280	0.001
Catalase, U/mg	*p* = 0.52 r = 0.12	*p* = 0.45 r = 0.14	*p* = 0.81 r = 0.04	0.826
Malondialdehyde (MDA), nmol/mg	*p* = 0.618 r = 0.09	*p* = 0.034 * r = 0.39	*p* = 0.009 * r = 0.48	0.021
Diene conjugates (DC), U/mg	*p* = 0.004 * r = 0.52	*p* = 0.013 * r = 0.53	*p* = 0.575 r = 0.11	0.004

Note: “*” indicates statistically significant differences at *p* < 0.05.

**Table 3:** Effect of fortified curd product on levels of immunoglobulins and proinflammatory cytokines in rats (in vivo).

Indicator	Control Group (Me; Q1–Q3)	Experimental Group 1 (Me; Q1–Q3)	Experimental Group 2 (Me; Q1–Q3)	*p*-Value
IgA, units/mL	3799 (3040–4375)	2734 (1890–3199)	3554 (2722–4069)	0.006
IgM, units/mL	2444 (1718–3213)	3620 (1973–4940)	2578 (1462–4749)	0.159
IgG, units/mL	1.114 (0.495–2.837)	2476 (2098–4204)	2.332 (0.742–4.024)	0.097
TNF-α, pg/mL	4269 (3698–4622)	4327 (3700–4597)	4312 (3659–4601)	0.978
IL-6, pg/mL	4514 (4036–4623)	4215 (3444–4559)	3597 (3456–4201)	0.079

**Table 4:** Effect of the enriched curd product on immunoglobulin and proinflammatory cytokine levels in rats (in vivo): intergroup comparison.

Parameter	Control Group—Experimental Group 1	Control Group—Experimental Group 2	Experimental Group 1—Experimental Group 2
IgA, U/mL	*p* = 0.01 * r = 0.43	*p* = 0.84 r = 0.20	*p* = 0.56 r = 0.02
IgM, U/mL	*p* = 0.03 * r = 0.47	*p* = 0.09 r = 0.40	*p* = 0.15 r = 0.36
IgG, U/mL	*p* = 0.024 * r = 0.48	*p* = 0.135 r = 0.36	*p* = 0.90 r = 0.19
TNF-α, pg/mL	*p* = 0.12 r = 0.37	*p* = 0.75 r = 0.21	*p* = 0.64 r = 0.08
IL-6, pg/mL	*p* = 0.83 r = 0.04	*p* = 0.91 r = 0.02	*p* = 0.74 r = 0.06

Note: “*”—statistically significant at *p* < 0.05.

**Table 5:** Indicators of enzymatic activity of purine metabolism in rats when introducing a fortified curd product into the diet (in vivo).

Indicator	Control Group (Me; Q1–Q3)	Experimental Group 1 (Me; Q1–Q3)	Experimental Group 2 (Me; Q1–Q3)	*p*-Value
5’-nucleotidase activity	31,944 (26,620–53,241)	47,259 (30,671–57,659)	19,097 (15,046–25,463)	0.001
Adenosine deaminase activity	274,306 (142,361–722,569)	432,528 (320,015–530,478)	348,958 (260,417–619,792)	0.553
AMP deaminase activity	269,097 (79,861–443,750)	215,278 (160,547–326,389)	303,819 (163,542–468,750)	0.330

Note: Units of measurement for all parameters are nmol NH_3_/min/mg protein.

## Data Availability

The original contributions presented in this study are included in the article. Further inquiries can be directed to the corresponding authors.
